# Feline leptospirosis prevalence worldwide: A systematic review and meta-analysis of diagnostic approaches

**DOI:** 10.14202/vetworld.2024.255-272

**Published:** 2024-02-01

**Authors:** Morsid Andityas, Dian Meididewi Nuraini, Pornphutthachat Sota, Shih Keng Loong, Banchob Sripa, Peerapol Sukon, Prasarn Tangkawattana, Sirikachorn Tangkawattana

**Affiliations:** 1 Veterinary Science Program, Faculty of Veterinary Medicine, Khon Kaen University, Khon Kaen, 40002, Thailand; 2 Veterinary Technology Study Program, Department of Bioresources Technology and Veterinary, Vocational College, Universitas Gadjah Mada, 55281, Indonesia; 3 Department of Animal Science, Faculty of Animal Science, Sebelas Maret University, Surakarta, 57126, Indonesia; 4 Tropical Disease Research Center, WHO Collaborating Center for Research and Control of Opisthorchiasis (Southeast Asian Liver Fluke Disease), Khon Kaen University, Khon Kaen, 40002, Thailand; 5 Tropical Infectious Diseases Research and Education Centre, Higher Institution Centre of Excellence, Universiti Malaya, Kuala Lumpur, 50603, Malaysia; 6 Department of Tropical Medicine, Faculty of Medicine, Khon Kaen University, Khon Kaen, 40002, Thailand; 7 Department of Veterinary Anatomy, Faculty of Veterinary Medicine, Khon Kaen University, Khon Kaen, 40002, Thailand; 8 Department of Veterinary Pathobiology, Faculty of Veterinary Medicine, Khon Kaen University, Khon Kaen, 40002, Thailand

**Keywords:** cat, diagnosis, felid, global prevalence, *Leptospira* spp, meta-analysis, natural infection

## Abstract

**Background and Aim:**

Leptospirosis in felids (domestic and wild cats) presents an ongoing challenge in our understanding. Numerous studies have reported the detection of *Leptospira* spp. in these feline populations, highlighting their potential as zoonotic carriers. This systematic review and meta-analysis aimed to provide insight into the global prevalence of leptospirosis in domestic and wild cats.

**Materials and Methods:**

We conducted extensive searches across five databases (PubMed, Scopus, Web of Science, Science Direct, and Google Scholar) following the Preferred Reporting Items for Systematic Reviews and Meta-analyses Protocols guidelines. Random-effect meta-analyses were performed using R software version 4.3.0 to estimate pooled prevalence rates. Subgroup meta-analyses were conducted based on continents, diagnostic methods, sample types, and wildcat genera.

**Results:**

A total of 71 articles on leptospirosis in domestic cats and 23 articles on leptospirosis in wild cats met the eligibility criteria. Our findings indicated a significantly higher pooled seroprevalence of leptospirosis in domestic cats compared with infection prevalence (9.95% [95% confidence interval (CI), 7.60%–12.54%] vs. 4.62% [95% CI, 2.10%–7.83%], p = 0.01). In contrast, no significant difference was observed in pooled seroprevalence and infection prevalence among wild cats (13.38% [95% CI, 6.25%–21.93%] vs. 2.9% [95% CI, 0.00%-18.91%], p = 0.21). A subgroup meta-analysis of domestic cats revealed significant differences in seroprevalence across continents, sample types, and diagnostic methods. On the contrary, wild cats had no significant differences in any of the subgroups.

**Conclusion:**

*Leptospira* spp. have evidently been exposed to both domestic and wild cats, highlighting their potential roles as reservoir hosts for leptospirosis. These findings highlight the importance of considering felids as a possible public health threat.

## Introduction

Leptospirosis is one of the most widespread zoonotic and waterborne diseases on a global scale [1]. This infectious ailment affects a broad spectrum of animals, including rats, horses, cows, pigs, dogs, sea lions, and even felids such as cats [2]. However, the symptoms of leptospirosis are rarely observed in cats, with clinical manifestations predominantly observed in young cats [2]. It has been postulated that cats may show resistance to leptospirosis, particularly in their propensity for rodent predation, even though there is a lower likelihood of developing clinical symptoms of leptospirosis. This suggested resistance is also linked to the acidity level of cat urine, purportedly diminishing the viability of *Leptospira* spp. [3, 4]. Although clinical signs of *Leptospira* spp. exposure are infrequently exhibited in cats, serological evidence of *Leptospira* spp. exposure has been documented in domestic cats (*Felis catus*) presenting sub-clinical symptoms and apparently healthy cats [5]. This raises concerns about the potential transmission of *Leptospira* spp. from cats to the surrounding environment [5]. However, the precise role of cats in leptospirosis pathogenesis still needs to be further understood [2]. Cats may act as reservoir hosts for leptospirosis [2, 3, 6–9], indefinitely sustaining the circulation of infectious agents in the environment and serving as a transmission source to other animals. Due to the absence of clinical symptoms in many cats, they are also called carrier hosts [2, 10, 11].

Notably, leptospirosis has been observed not only in domestic cats but also in various wild feline species, such as jaguars (*Panthera onca*), mountain lions (*Puma concolor*), and bobcats (*Lynx rufus*) [12, 13]. These findings may raise public health concerns, especially as human activities encroached on wildlife habitats, leading to increased interactions between animals and humans, thereby amplifying the potential for pathogen transmission [14]. In addition, some wild animals reside in captivity within zoological parks and circuses situated near urban areas, which further increases the risk of human exposure to zoonotic agents, including *Leptospira* spp. [15, 16].

To date, many epidemiological studies employing various diagnostic methods have been undertaken to unravel the prevalence of leptospirosis in felids [2, 17–21]. However, the results of these studies show varying prevalence across different regions globally. In addition, recent systematic reviews and meta-analyses have mainly focused on elucidating the prevalence of leptospirosis in domestic cats [22, 23]. Consequently, this research endeavors to provide a comprehensive investigation into the seroprevalence and infection prevalence of leptospirosis in both domestic and wild cats using various diagnostic methodologies. This systematic review and meta-analysis aimed to provide insight into the global prevalence of leptospirosis in domestic and wild cats.

## Materials and Methods

### Ethical approval

This study was conducted according to the Preferred Reporting Items for Systematic Reviews and Meta-Analyses guidelines [24]. The protocol has been published in Systematic Reviews for Animals and Food (SYREAF; https***://***syreaf.org***/***protocols/) and OSF (https://osf.io/b3u8j/).

### Study period and location

This systematic review and meta-analysis was conducted from October 21, 2022, to May 9, 2023. The data were extracted at Faculty of Veterinary Medicine, Khon Kaen University, Thailand.

### Search strategy

The search strategy comprehensively explored five important databases: PubMed, Scopus, Web of Science, Science Direct, and Google Scholar. MeSH terms related to “*Leptospira*,” “domestic and wild feline,” “prevalence,” and “diagnostic”: These terms were systematically combined or separated within the search engine using “AND” and “OR” as keyword string (https://osf.io/sym2a).

### Selection criteria

All extracted articles underwent a comprehensive evaluation using the Rayyan-Intelligent Systematic Review platform (www.rayyan.ai) to check for duplication and screen the articles to ensure the integrity of the research process. Two reviewers, MA and DMN within the Rayyan platform, independently selected the manuscript through title and abstract screening. The screening process was performed on the basis of several inclusion criteria, such as a study population consisting of both domestic and wild cats, and the outcomes should specifically report the prevalence of leptospirosis. Case studies, surveys, or cross-sectional studies were eligible for inclusion. However, articles reporting experimental studies, cohort studies, or case–control studies were excluded from consideration. In addition to these criteria, articles were included only if they provided detailed information on sample sizes for each feline species, types of samples collected, and diagnostic methods. The precise questions used as screening guidelines have been described in the protocol.

### Data extraction

Data were extracted by MA and subsequently reviewed by DMN. Inconsistencies or discrepancies that arose during this process have been carefully resolved through discussion and consensus. For inclusion in the meta-analysis, articles were required to provide comprehensive information regarding sample sizes for each feline species, types of samples collected, and diagnostic methods employed. The extracted data encompassed essential details, including author names, publication years, study locations (both country and continent)**,** animal species involved, sample sizes and types, method of detection, and number of leptospirosis cases identified. All extracted data were collated into pre-designed Microsoft Excel sheets (Microsoft Corp., Redmond, WA, USA).

### Quality assessment of individual studies

The quality assessment was performed using the Joanna Briggs Institute Critical Appraisal Checklist for studies reporting prevalence data [25]. In response to the specific criteria outlined in the checklist, each study’s quality was categorized as “yes,” “no,” “unclear,” or “not applicable.” The overall results were subsequently classified into three tiers based on the scores obtained***:*** high (***≥***7), medium (4–6), and poor (***≤***3). This rigorous quality assessment process ensured that only studies meeting stringent quality criteria were included in our meta-analysis.

### Statistical analysis

Statistical analysis was conducted using the “Meta” and “Metaphor” packages in R 4.3.0 software (Comprehensive R Archive Network, Vienna, Austria) [26]. The Freeman–Tukey double arcsine transformation was applied to test statistical significance. Effect sizes were evaluated based on pooled prevalence and 95% confidence interval (CI). Heterogeneity was assessed using the I^2^ index, Cochrane’s Q test, and the corresponding p-value. When moderate or high heterogeneity (I^2^ > 50%)was detected, a random-effects model was employed, whereas a fixed-effects model was chosen in the presence of low heterogeneity.

The pooled prevalence analysis yielded seroprevalence and infection prevalence. Seroprevalence indicates indirect detection by measuring the presence of antibodies in cats, whereas infection prevalence indicates direct isolation and detection of *Leptospira* spp. from samples. In addition, the analysis was stratified to differentiate between domestic and wildcat populations due to lifestyle, environment, and disease exposure. This division allowed for a clearer understanding of the prevalence of leptospirosis.

Subgroup meta-analyses were performed based on continent, sample type, diagnostic method, and infection status. The genus was used for subgroup meta-analysis in the case of wild cats. We performed a meta-regression and cumulative meta-analysis to assess the trends of leptospirosis in domestic and wild cats based on publication years. Sensitivity analysis was also performed to verify the robustness of pooled prevalence in domestic and wild cats using leave-one-out meta-analysis, which indicates the disproportional influence of each study on the results. The Egger’s test and funnel plot [27] were used to determine publication bias. The global distribution of leptospirosis in cats was displayed using QGIS v.3.2.0 (https://qgis.org/en/site/).

## Results

### Literature search

A total of 1387 studies were initially identified for inclusion in this systematic review and meta-analysis. After the initial screening procedure, 124 articles met the eligibility criteria and progressed to further evaluation. Of these, 32 articles were subsequently excluded mainly due to unavailability of full-text access (n = 26) and inappropriate outcome measures (n = 6). As a result, a total of 91 studies were ultimately included in the meta-analysis.

**Figure-1:**
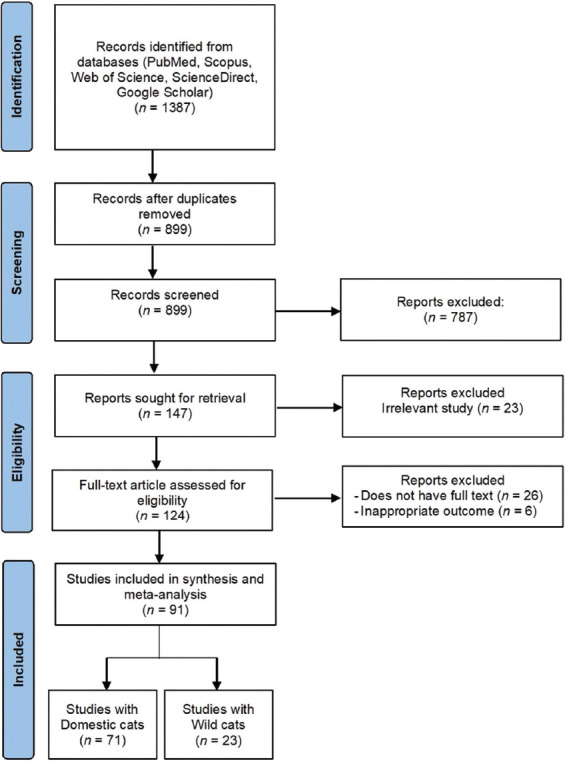
PRISMA flow diagram of selected articles included in this study.

The dataset used for the meta-analysis comprised 13,034 samples collected from domestic cats in 71 studies and 1034 samples collected from wild cats in 23 studies (Tables-1 and 2) [3, 5–13, 16–21, 28–101]. Figure-1 illustrates the detailed process of study retrieval, screening, and collating studies on the prevalence of leptospirosis in domestic and wild cats.

**Table-1:** Characteristics of the eligible studies based on the seroprevalence and infection prevalence of leptospirosis in domestic and stray cats.

Location	Period	Domestic/stray	Test	Cut-off	Total	Event	Positive (%)	Status	Sample	Reference
Africa										
Egypt	2006–2007	Stray	MAT	1:50	2	1	50.0	Seroprevalence	Serum	Felt *et al*. [8]
Egypt	2006–2007	Stray	PCR	-	2	1	50.0	Infection Prevalence	Kidney	Felt *et al*. [8]
Algeria	2017	Stray	PCR and qPCR	-	107	0	0.0	Infection Prevalence	Urine	Zaidi *et al*. [33]
Botswana	2009–2014	Domestic	PCR	-	4	0	0.0	Infection Prevalence	Kidney	Jobbins and Alexander [34]
Algeria	ns	Stray	MAT	1:100	144	8	5.6	Seroprevalence	Serum	Zaidi *et al*. [35]
ASIA										
Japan	2012–2018	Stray	MAT	160	241	40	16.6	Seroprevalence	Serum	Kakita *et al*. [36]
Japan	2012–2018	Stray	Nested PCR	-	42	3	7.1	Infection Prevalence	Urine	Kakita *et al*. [36]
Thailand	2016–2017	Domestic	MAT	1:20	260	14	5.4	Seroprevalence	Serum	Sprißler *et al*. [6]
Thailand	2016–2017	Domestic	qPCR	-	260	2	0.8	Infection Prevalence	Urine	Sprißler *et al*. [6]
Thailand	2016–2017	Domestic	Culture	-	260	0	0.0	Infection Prevalence	Urine	Sprißler *et al*. [6]
Malaysia	2012–2013	Stray	PCR	-	14	0	0.0	Infection Prevalence	Kidney	Benacer *et al*. [37]
Malaysia	2012–2013	Stray	Culture/DFM	-	14	0	0.0	Infection Prevalence	Kidney	Benacer *et al*. [37]
Malaysia	2012–2013	Stray	PCR	-	36	0	0.0	Infection Prevalence	Urine	Benacer *et al*. [37]
Malaysia	2012–2013	Stray	Culture/DFM	-	36	0	0.0	Infection Prevalence	Urine	Benacer *et al*. [37]
Malaysia	2017–2018	Domestic	MAT	≥100	82	21	25.6	Seroprevalence	Serum	Alashraf *et al*. [5]
Malaysia	2017–2018	Domestic	PCR	-	82	4	4.9	Infection Prevalence	Urine	Alashraf *et al*. [5]
Malaysia	2017–2018	Domestic	PCR	-	87	7	8.0	Infection Prevalence	Blood	Alashraf *et al*. [5]
Malaysia	2017–2018	Domestic	Culture	-	82	4	4.9	Infection Prevalence	Kidney	Alashraf *et al*. [5]
Malaysia	2017–2018	Domestic	Culture	-	82	1	1.2	Infection Prevalence	Urine	Alashraf *et al*. [5]
Malaysia	2017–2018	Shelter	MAT	≥1:100	110	20	18.2	Seroprevalence	Serum	Alashraf *et al*. [38]
South Korea	2008	Stray	PCR		24	15	62.5	Infection Prevalence	Kidney	Truong *et al*. [39]
Iran	2008–2012	Stray and domestic	MAT	≥1:100	147	19	12.9	Seroprevalence	Serum	Garoussi *et al*. [40]
Malaysia	ns	Shelter	MAT	≥1:100	47	7	14.9	Seroprevalence	Serum	Alashraf *et al*. [41]
Vietnam	2019	ns	MAT	1:100	164	20	12.2	Seroprevalence	Serum	Mai *et al*. [42]
Iran	2009	Domestic	PCR	-	132	28	21.2	Infection Prevalence	Blood	Azizi *et al*. [43]
Indonesian	ns	Stray and domestic	MAT	≥1:100	27	1	3.7	Seroprevalence	Serum	Mulyani *et al*. [44]
Iran	2003	Stray and domestic	MAT	1:100	111	30	27.0	Seroprevalence	Serum	Jamshidi *et al*. [45]
Iran	2007–2008	Stray	MAT	≥1:100	102	5	4.9	Seroprevalence	Serum	Mosallanejad *et al*. [46]
Iran	2011	Stray and domestic	MAT	1:50	71	1	1.4	Seroprevalence	Serum	Khomayezi *et al*. [47]
Japan	1999–2001	Domestic	MAT	ns	117	9	7.7	Seroprevalence	Serum	Akuzawa *et al*. [48]
Taiwan	2010–2011	Stray and domestic	MAT	1:100	225	21	9.3	Seroprevalence	Serum	Chan *et al*. [7]
Taiwan	2010–2011	Stray and domestic	PCR	-	131	25	19.1	Infection Prevalence	Serum	Chan *et al*. [7]
Taiwan	2010–2011	Stray and domestic	PCR	-	118	80	67.8	Infection Prevalence	Urine	Chan *et al*. [7]
Thailand	2014–2018	Stray	LLT	-	64	7	10.9	Seroprevalence	Serum	Ngasaman *et al*. [49]
Thailand	2014–2018	Stray	PCR	-	64	5	7.8	Infection Prevalence	Blood	Ngasaman *et al*. [49]
Australia										
New Caledonia	2009	Domestic	MAT	1:40	8	8	100.0	Seroprevalence	Serum	Roqueplo *et al*. [50]
New Zeland	ns	Domestic	MAT	1:24	225	20	8.9	Seroprevalence	Serum	Shophet [51]
Australia	2011–2013	Stray	PCR	-	141	25	17.7	Infection Prevalence	Kidney	Dybing *et al*. [9]
Australia	1988–1990	Domestic	MAT	1:50	59	10	16.9	Seroprevalence	Serum	Dickeson and Love [52]
Europe										
Spain	2004–2007	Stray	MAT	1:100	44	6	13.6	Seroprevalence	Serum	Millán *et al*. [53]
Spain	2004–2007	Stray	SSM, IM and Culture	ns	25	5	20.0	Infection Prevalence	Urine and kidney	Millán *et al*. [53]
France	2013	Stray and domestic	MAT	1:40	92	34	37.0	Seroprevalence	Serum	Holzapfel *et al*. [32]
France	2013	Stray and domestic	qPCR	-	45	6	13.3	Infection Prevalence	Kidney	Holzapfel *et al*. [32]
France	2013	Stray and domestic	qPCR	-	89	4	4.5	Infection Prevalence	Urine	Holzapfel *et al*. [32]
France	2013	Stray and domestic	qPCR	-	78	5	6.4	Infection Prevalence	Blood	Holzapfel *et al*. [32]
Estonia	2013 and 2015	Domestic	MAT	≥100	546	70	12.8	Seroprevalence	Serum	Lehtla *et al*. [54]
Italy	2014–2016	Stray	MAT	1:100	99	10	10.5	Seroprevalence	Serum	Mazzotta *et al*. [55]
Italy	2014–2016	Stray	qPCR	-	99	1	1.1	Infection Prevalence	Urine	Mazzotta *et al*. [55]
Italy	2014–2016	Stray	qPCR	-	99	0	0	Infection Prevalence	Blood	Mazzotta *et al*. [55]
Spain	2017–2018	Stray and domestic	MAT	1:20	244	10	4.1	Seroprevalence	Serum	Murillo *et al*. [56]
Spain	2017–2018	Stray and domestic	qPCR	-	232	4	1.7	Infection Prevalence	Urine	Murillo *et al*. [56]
Spain	2017–2018	Stray and domestic	qPCR	-	89	1	1.1	Infection Prevalence	Blood	Murillo *et al*. [56]
Czech Republic	2013–2015	Domestic	MAT	1:100	360	33	9.2	Seroprevalence	Serum	Zakovska *et al*. [57]
France	2009	Stray	MAT	1:100	30	8	26.7	Seroprevalence	Serum	Desvars *et al*. [58]
France	2009	Stray	qPCR	-	21	6	28.6	Infection Prevalence	Kidney	Desvars *et al*. [58]
France	2015–2016	Stray	qPCR	-	172	1	0.6	Infection Prevalence	Kidney	Gomard *et al*. [59]
Germany	2013–2015	Domestic	MAT	1:≥100	195	35	17.9	Seroprevalence	Serum	Weis *et al*. [60]
Germany	2013–2015	Domestic	qPCR	-	215	7	3.3	Infection Prevalence	Urine	Weis *et al*. [60]
Greece	1997–1998	Domestic	MAT	≥1:50	99	33	33.3	Seroprevalence	Serum	Mylonakis *et al*. [61]
Italy	2018–2019	Domestic	MAT	≥1:20	111	17	15.3	Seroprevalence	Serum	Donato *et al*. [62]
Italy	2018–2019	Domestic	qPCR	-	111	4	3.6	Infection Prevalence	Blood	Donato *et al*. [62]
Italy	2018–2019	Domestic	qPCR	-	111	10	9.0	Infection Prevalence	Urine	Donato *et al*. [62]
Switzerland	2017	Domestic	MAT	≥100	107	11	10.3	Seroprevalence	Serum	Hässle *et al*. [30]
Germany	2012–2016	Domestic	MAT	≥1:100	175	28	16.0	Seroprevalence	Serum	Rose *et al*. [29]
Spain	2011–2013	Stray	PCR	-	27	2	7.4	Infection Prevalence	Urine and kidney	Millán *et al*. [63]
Serbia	2012–2013	Stray	MAT	≥1:100	161	43	26.7	Seroprevalence	Serum	Sonja *et al*. [64]
Portugal	2014–2018	Domestic	ELISA	>1	243	23	9.5	Seroprevalence	Serum	Moreira da Silva *et al*. [65]
Italy	2017–2021	Stray	MAT	1:100	391	1	0.3	Seroprevalence	Serum	Grippi *et al*. [66]
Italy	2017–2021	Stray	qPCR	-	391	1	0.3	Infection Prevalence	Blood	Grippi *et al*. [66]
Italy	2017–2021	Stray	qPCR	-	32	2	6.3	Infection Prevalence	Urine	Grippi *et al*. [66]
Scotland	ns	Domestic	MAT	1:30	87	8	9.2	Seroprevalence	Serum	Agunloye and Nash [67]
Bulgaria	ns	Domestic	MAT	ns	65	24	36.9	Seroprevalence	Serum	Stoichev *et al*. [68]
North America										
Canada	2017–2018	Stray	MAT	1:50	200	20	10.0	Seroprevalence	Serum	Bourassi *et al*. [31]
Canada	2017–2018	Stray	PCR	-	200	7	3.5	Infection Prevalence	Urine	Bourassi *et al*. [31]
United States	2017 and 2020	Stray	qPCR	-	52	0	0.0	Infection Prevalence	Blood and Urine	Sebastian *et al*. [69]
United States	2018 and 2020	Stray	MAT	1:100	127	17	13.4	Seroprevalence	Serum	Sebastian *et al*. [69]
America	2008–2009	Stray	MAT	≥1:25	18	0	0.0	Seroprevalence	Serum	Grimm *et al*. [70]
Canada	2007	Domestic	MAT	≥1:100	40	10	25.0	Seroprevalence	Serum	Lapointe *et al*. [71]
America	2010	Stray	MAT	≥1:100	63	3	4.8	Seroprevalence	Serum	Markovich *et al*. [72]
America	ns	Domestic	MAT	≥1:100	350	17	4.9	Seroprevalence	Serum	Murphy *et al*. [73]
Saint Kitts and Nevis	2014–2015	Domestic	MAT	1:100	50	2	4.0	Seroprevalence	Serum	Pratt *et al*. [74]
St. Kitts	2015	Stray	MAT	1:100	102	7	6.9	Seroprevalence	Serum	Betance *et al*. [10]
St. Kitts	2015	Stray	PCR	-	103	1	1.0	Infection Prevalence	Urine	Betance *et al*. [10]
Canada	2010–2012	Domestic	MAT	≥1:100	239	26	10.9	Seroprevalence	Serum	Rodriguez *et al*. [11]
Canada	2010–2012	Domestic	PCR		238	8	3.4	Infection Prevalence	Urine	Rodriguez *et al*. [11]
United States	2017–2018	Domestic	MAT	1:100	43	0	0.0	Seroprevalence	Serum	Spangler *et al*. [75]
United States	2017–2018	Domestic	qPCR	-	46	0	0.0	Infection Prevalence	Urine	Spangler *et al*. [75]
United States	2015–2016	Stray and domestic	MAT	1:100	139	12	8.6	Seroprevalence	Serum	Palerme *et al*. [76]
South America										
Brazil	2008–2010	Domestic	MAT	ns	29	1	3.4	Seroprevalence	Serum	Furtado *et al*. [17]
Chile	2016–2017	Domestic	qPCR	-	231	30	13.0	Infection Prevalence	Urine	Dorsch *et al*. [3]
Chile	2016–2017	Domestic	Culture	-	231	7	3.0	Infection Prevalence	Urine	Dorsch *et al*. [3]
Brazil	2020–2021	Domestic	MAT	≥100	39	5	12.8	Seroprevalence	Serum	Pinheiro *et al*. [77]
Brazil	2014–2015	Domestic	MAT	ns	10	0	0.0	Seroprevalence	Serum	de Souza Rocha *et al*. [78]
Brazil	2014–2015	Domestic	PCR	-	10	0	0.0	Infection Prevalence	Blood	de Souza Rocha *et al*. [78]
Colombia	2018	Domestic	MAT	ns	32	0	0.0	Seroprevalence	Serum	Molina *et al*. [79]
Argentina	2007	Domestic	MAT	≥100	5	0	0.0	Seroprevalence	Serum	Uhart *et al*. [80]
Brazil	ns	Stray	MAT	1:100	200	0	0.0	Seroprevalence	Serum	de Morais *et al*. [81]
Brazil	2013	Domestic	MAT	1:100	43	2	4.7	Seroprevalence	Serum	Dos Santos *et al*. [82]
Mexico	2015	Domestic	MAT	1:100	13	2	15.4	Seroprevalence	Serum	Ortega-Pacheco *et al*. [83]
Brazil	2016	Domestic	MAT	1:50	65	3	4.6	Seroprevalence	Serum	Cordeiro *et al*. [84]
Brazil	2016	Domestic	PCR	-	65	1	1.5	Infection Prevalence	Urine	Cordeiro *et al*. [84]
Chile	2011–2012	Domestic	MAT	1:100	124	10	8.1	Seroprevalence	Serum	Azócar-Aedo *et al*. [28]
Brazil	2011	Stray & domestic	MAT	1:100	129	7	5.4	Seroprevalence	Serum	Brasil *et al*. [85]
Brazil	2015–2018	Stray & domestic	MAT	1:100	180	10	5.6	Seroprevalence	Serum	Ribeiro *et al*. [86]
Brazil	2008	Domestic	MAT	1:100	330	23	7.0	Seroprevalence	Serum	Parreira *et al*. [87]
Mexico	ns	Domestic	MAT	ns	260	46	17.7	Seroprevalence	Serum	Ortega-Pacheco *et al*. [88]
Brazil	2007–2014	Stray	MAT	≥1:100	220	11	5.0	Seroprevalence	Serum	Silva *et al*. [89]

ELISA=Enzyme-linked immunosorbent assay, LLT=Lepto-latex test, MAT=Microscopic agglutination test, ns=Not specified, PCR=Polymerase chain reaction, qPCR=Quantitative polymerase chain reaction, DFM=Dark field microscopy, SSM=Silver staining method, IM=Immunofluorescence

**Table-2:** Characteristics of the eligible studies based on the seroprevalence and infection prevalence of leptospirosis in wild cats.

Country	Period	Host Species	Test	Cut-off	Total	Event	Positive (%)	Status	Sample	Author
Africa										
Tanzania	2012–2013	Lion	MAT	≥1:160	2	1	50.00	Seroprevalence	Serum	Assenga *et al*. [90]
Botswana	2009–2014	Lion	PCR	-	1	0	0.00	Infection Prevalence	Kidney	Jobbins and Alexander [34]
Asia										
Nepal	2011–2017	Bengal tigers *(Panthera tigris tigris)*	MAT	>100	11	6	54.55	Seroprevalence	Serum	McCauley *et al*. [21]
Laos	2014–2017	Cat *(Prionailurus bengalensis)*	PCR	-	3	1	33.3	Infection Prevalence	ns	Nawtaisong *et al*. [91]
Laos	2014–2017	Cat *(Catopuma temminckii)*	PCR	-	1	1	100.0	Infection Prevalence	ns	Nawtaisong *et al*. [91]
Mongolia	2008–2015	Snow leopards *(Panthera unica)*	ELISA	ns	20	4	20.00	Seroprevalence	Serum	Esson *et al*. [19]
Mongolia	2008–2015	Snow leopards *(Panthera unica)*	MAT	≥100	20	2	10.00	Seroprevalence	Serum	Esson *et al*. [19]
South Korea	2017–2019	Leopard cat *(Prionailurus bengalensis)*	Nested PCR	-	22	0	0.00	Infection Prevalence	Feces	Kumari *et al*. [92]
China	2018–2019	Tigers	PCR	-	119	0	0.00	Infection Prevalence	Blood	Xianglei *et al*. [93]
China	2018–2019	Tigers	ELISA	PC	119	50	42.02	Seroprevalence	Serum	Xianglei *et al*. [93]
Europe										
Slovenia	2019–2020	Eurasian lynx *(Lynx lynx)*	MAT	≥50	2	2	100.0	Seroprevalence	Serum	Žele-Vengušt *et al*. [94]
Spain	2004–2007	Iberian lynx *(Lynx pardinus)*	MAT	1:100	22	7	31.82	Seroprevalence	Serum	Millán *et al*. [53]
Spain	2004–2007	Iberian lynx *(Lynx pardinus)*	IM	-	9	1	11.11	Infection Prevalence	Kidney and urine	Millán *et al*. [53]
Italy	2019	Tigers	MAT	1:100	20	3	15.00	Seroprevalence	Serum	Iatta *et al*. [16]
Spain	2011–2013	Wildcats *(Felis silvestris silvestris)*	MAT	ns	9	3	33.33	Seroprevalence	Serum	Candela *et al*. [95]
North America										
America	2007–2017	Bobcat (*Lynx rufus)*	qPCR	-	34	3	8.82	Infection Prevalence	Kidney and urine	Straub and Foley [12]
America	2007–2017	Mountain lion *(Puma concolor)*	qPCR	-	119	28	23.53	Infection Prevalence	Kidney and urine	Straub and Foley [12]
America	2007–2017	Bobcat *(Lynx rufus)*	MAT	≥100	31	10	32.26	Seroprevalence	Serum	Straub and Foley [12]
America	2007–2017	Mountain lion *(Puma concolor)*	MAT	≥100	127	63	49.61	Seroprevalence	Serum	Straub and Foley [12]
America	1992–1995	Bobcat *(Lynx rufus)*	MAT	-	25	0	0.00	Seroprevalence	Serum	Riley *et al*. [96]
Canada	1997–1998	Wild lynx *(Lynx canadensis)*	MAT	≥1:100 & 1:50	97	1	1.03	Seroprevalence	Serum	Labelle *et al*. [97]
Canada	1997–1998	Bobcat *(Lynx rufus)*	MAT	≥1:100 & 1:50	11	1	9.09	Seroprevalence	Serum	Labelle *et al*. [97]
America	1982–1984	Bobcat *(Lynx rufus)*	MAT	ns	8	2	25.00	Seroprevalence	Serum	Heidt *et al*. [98]
South America										
Brazil	2006	Geoffroy’s cat *(Leopardus geoffroyi)*	MAT	100	1	0	0.00	Seroprevalence	Serum	Ullmann *et al*. [18]
Brazil	2006	Jaguarundis *(Puma yagouaroundi)*	MAT	100	3	0	0.00	Seroprevalence	Serum	Ullmann *et al*. [18]
Brazil	2006	Margays *(Leopardus wiedii)*	MAT	100	17	1	5.88	Seroprevalence	Serum	Ullmann *et al*. [18]
Brazil	2006	Little spotted cats *(Leopardus tigrinus)*	MAT	100	22	0	0.00	Seroprevalence	Serum	Ullmann *et al*. [18]
Brazil	2006	Ocelots *(Leopardus pardalis)*	MAT	100	14	1	7.14	Seroprevalence	Serum	Ullmann *et al*. [18]
Brazil	2000–2009	Jaguars *(Panthera onca)*	MAT	ns	31	13	41.94	Seroprevalence	Serum	Furtado *et al*. [17]
Argentina	2000–2008	Geoffroy’s cat *(Leopardus geoffroyi)*	MAT	≥100	40	15	37.50	Seroprevalence	Serum	Uhart *et al*. [80]
Brazil	2010–2012	Jaguars *(Panthera onca)*	MAT	1:100	11	2	18.18	Seroprevalence	Serum	Onuma *et al*. [13]
Brazil	2008	Puma *(Puma concolor)*	MAT	1:100	3	0	0.00	Seroprevalence	Serum	Pimentel *et al*. [99]
Brazil	2008	Jaguar *(Panthera onca)*	MAT	1:100	1	0	0.00	Seroprevalence	Serum	Pimentel *et al*. [99]
Brazil	ns	Chaus cat *(Felis chaus)*	MAT	1:50	3	0	0.00	Seroprevalence	Serum	Lilenbaum *et al*. [20]
Brazil	ns	Linx *(Felis lynx lynx)*	MAT	1:50	1	0	0.00	Seroprevalence	Serum	Lilenbaum *et al*. [20]
Brazil	ns	Serval *(Felix serval)*	MAT	1:50	2	0	0.00	Seroprevalence	Serum	Lilenbaum *et al*. [20]
Brazil	ns	Yagouaroundi *(Herpailurus yagouaroundi)*	MAT	1:50	2	1	50.00	Seroprevalence	Serum	Lilenbaum *et al*. [20]
Brazil	ns	Ocelot *(Leopardus pardalis)*	MAT	1:50	2	0	0.00	Seroprevalence	Serum	Lilenbaum *et al*. [20]
Brazil	ns	Spotted cat *(Leopardus tigrina)*	MAT	1:50	2	0	0.00	Seroprevalence	Serum	Lilenbaum *et al*. [20]
Brazil	ns	Margay (*Leopardus wiedii)*	MAT	1:50	3	1	33.33	Seroprevalence	Serum	Lilenbaum *et al*. [20]
Brazil	ns	Lion *(Panthera leo)*	MAT	1:50	5	0	0.00	Seroprevalence	Serum	Lilenbaum *et al*. [20]
Brazil	ns	Jaguar *(Panthera onca)*	MAT	1:50	2	1	50.00	Seroprevalence	Serum	Lilenbaum *et al*. [20]
Brazil	ns	Black Jaguar *(Panthera onca var*. *melanica)*	MAT	1:50	1	0	0.00	Seroprevalence	Serum	Lilenbaum *et al*. [20]
Brazil	ns	Leopard *(Panthera pardus)*	MAT	1:50	2	0	0.00	Seroprevalence	Serum	Lilenbaum *et al*. [20]
Brazil	ns	Tiger *(Panthera tigris)*	MAT	1:50	3	0	0.00	Seroprevalence	Serum	Lilenbaum *et al*. [20]
Brazil	ns	Mountain lion *(Puma concolor)*	MAT	1:50	2	1	50.00	Seroprevalence	Serum	Lilenbaum *et al*. [20]
Bolivia	2001–2005	Ocelot *(Leopardus pardalis)*	MAT	1:100	8	0	0.0	Seroprevalence	Serum	Fiorello *et al*. [100]
Bolivia	2001–2005	Geoffroy’s cat *(Oncifelis geoffroyi)*	MAT	1:100	9	0	0.0	Seroprevalence	Serum	Fiorello *et al*. [100]
Bolivia	2001–2005	Jaguarundi *(Herpailurus yaguarondi)*	MAT	1:100	1	0	0.0	Seroprevalence	Serum	Fiorello *et al*. [100]
Brazil	2002–2006	Puma *(Puma concolor)*	MAT	≥1:100	7	2	28.6	Seroprevalence	Serum	Jorge *et al*. [101]
Brazil	2002–2006	Ocelots *(Leopardus pardalis)*	MAT	≥1:100	4	3	75.0	Seroprevalence	Serum	Jorge *et al*. [101]

ELISA=Enzyme-linked immunosorbent assay; MAT, Microscopic Agglutination Test; ns, not specified; PCR, Polymerase Chain Reaction; qPCR, quantitative Polymerase Chain Reaction; IM, Immunofluorescence; PC=positive control (≥0.7) and negative control (≤0.35)

### Overall pooled prevalence

Overall pooled seroprevalence in domestic cats was estimated to be 9.95% (95% CI, 7.60%–12.54%), whereas it was 13.38% (95% CI, 6.25%–21.93%) in wild cats. Domestic cats exhibited a infection prevalence rate of 4.62% (95% CI, 2.10%–7.83%), whereas wild cats showed a infection prevalence rate of 2.9% (95% CI, 0.00%–18.91%). Subgroup analysis was employed to overcome the high heterogeneity in each population. Table-3 shows the overall pooled leptospirosis prevalence and subgroup meta-analysis results in domestic and wild cats.

**Table-3:** Pooled prevalence and subgroup analyses of leptospirosis domestic and wild cats.

Categories	n	Prevalence		Heterogeneity			p-value subgroup
	
		Estimates	95% CI	I^2^	Q	p-value	
Domestic cat							
Pooled prevalence							
Seroprevalence	62	9.95	7.60–12.54	89%	568.52	<0.01	0.01
Infection prevalence	45	4.62	2.10–7.83	93%	626.52	<0.01	
Continent							
Seroprevalence							
Asia	14	11.37	7.72–15.59	82%	74.2	<0.01	0.01
Australia	3	40.59	0.00–97.38	95%	40.68	<0.01	
Europe	17	14.95	9.83–20.89	94%	282.26	<0.01	
North America	11	7.21	4.28–10.75	71%	34.66	<0.01	
South America	14	5.06	2.45–8.36	84%	83.1	<0.01	
Infection prevalence							
Africa	3	0.39	0.00–33.30	65%	5.79	0.06	0.19
Asia	16	8.09	1.83–17.52	96%	420.91	<0.01	
Europe	16	3.94	1.62–7.04	81%	79.69	<0.01	
North America	5	1.81	0.61–3.49	29%	5.6	0.23	
South America	4	3.9	0.16–10.65	86%	20.93	<0.01	
Sample type							
Serum	63	10.09	7.76–12.66	89%	578.41	<0.01	<0.01
Blood	9	3.77	0.64–8.68	91%	88.68	<0.01	
Urine	22	3.63	1.01–7.51	95%	382.27	<0.01	
Kidney	10	9.87	0.72–24.57	90%	91.62	<0.01	
Diagnostic method							
MAT	60	9.96	7.52–12.66	90%	568.3	<0.01	<0.01
PCR	38	5.52	2.23–9.08	94%	574.15	<0.01	
Culture	6	0.83	0.00–3.11	69%	16.27	<0.01	
Wild cat							
Pooled prevalence							
Seroprevalence	43	13.38	6.25–21.93	79%	201.59	<0.01	0.21
Infection prevalence	8	2.9	0.00–18.91	89%	61.65	<0.01	
Continent							
Seroprevalence							
Asia	4	29.96	12.83–50.26	76%	12.62	<0.01	0.24
Europe	4	29.67	10.62–52.21	57%	6.93	0.07	
North America	6	14.98	1.41–36.52	96%	113.99	<0.01	
South America	28	6.95	0.59–17.17	45%	49.11	<0.01	
Sample type							
Serum	39	13.38	6.25–21.93	79%	201.59	<0.01	0.83
Urine + kidney	3	15.68	6.17–27.93	50%	3.98	0.14	
Diagnostic method							
MAT	41	12.07	4.91–20.94	78%	180.54	<0.01	0.60
PCR	7	2.57	0.00–22.14	90%	61.18	<0.01	
Host genus							
*Leopardus* spp.	11	5.96	0.00–21.45	68%	31.68	<0.01	0.54
*Lynx* spp.	9	13.13	2.22–28.89	84%	49.04	<0.01	
*Panthera* spp.	16	12.79	2.02–28.08	88%	128.87	<0.01	
*Felis* spp.	3	14.22	0.00–49.67	0%	1.51	0.47	
*Puma* spp.	7	23.96	7.05–44.81	74%	23.06	<0.01	

### Subgroup meta-analysis

#### Prevalence of leptospirosis in domestic cats: Analysis

The prevalence of leptospirosis in domestic cats was subjected to subgroup analysis based on continent, sample type, and diagnostic method. Significant differences were observed in seroprevalence (p = 0.01) across different continents as well as in seroprevalence between sample types (p < 0.01) and diagnostic methods (p < 0.01). However, there were no significant differences in infection prevalence among continents (p = 0.19).

**Figure-2:**
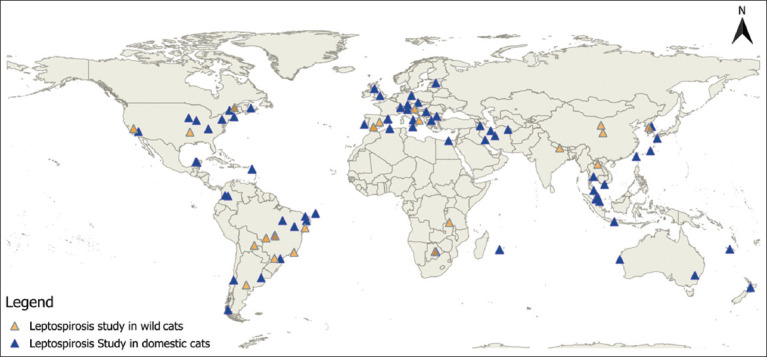
Distribution of cat leptospirosis studies worldwide. The distribution was visualized using basemap provided by QGIS software v.3.2.0.

Within the continent subgroup analysis, Australia reported the highest seroprevalence of leptospirosis in domestic cats (40.59%; 95% CI, 0.00%–97.38%), followed by Europe (14.95%; 95% CI, 9.83%–20.89%), Asia (11.37%; 95% CI, 7.72%–15.59%), North America (7.21%; 95% CI, 4.28%–10.75%), and South America (5.06%; 95% CI, 2.45%–8.36%). On the other hand, the highest infection prevalence among domestic cats was documented in Asia with a prevalence rate of 8.09% (95% CI, 1.83%–17.52%) while the lowest was recorded in Africa at 0.39% (95% CI, 0.00%–33.30%). An illustrative distribution of the prevalence of cat leptospirosis is presented in Figure-2.

Serum samples exhibited a higher prevalence of leptospirosis in domestic cats compared to urine samples, with rates of 10.09% (95% CI, 7.76%–12.66%) and 3.63% (95% CI, 1.01%–7.51%), respectively.

Microscopic agglutination test (MAT) emerged as the most frequently utilized diagnostic method in 60 studies, followed by a polymerase chain reaction (PCR) method in 38 studies and culture in six studies. Of these diagnostic methods, MAT had the highest prevalence at 9.96% (95% CI, 7.52%–22.66%), followed by PCR at 5.52% (95% CI, 2.23%–9.08%), and culture at 0.83% (95% CI, 0.00%–3.11%).

#### Analysis of leptospirosis prevalence in wild cats

Prevalence was also analyzed in subgroups of the continent, sample type, diagnostic method, and genus in wild cats. Notably, there were no significant differences in the results of these analyses among the subgroups.

Within the continent subgroup, the highest seroprevalence of leptospirosis in wild cats was identified in Asia at 29.96% (95% CI, 12.83%–50.26%), followed closely by Europe with a prevalence of 29.67% (95% CI, 10.62%–52.21%). North America reported a seroprevalence of 14.98% (95% CI, 1.41%–36.52%), whereas South America displayed a prevalence of 6.95% (95% CI, 0.59%–17.17%). However, due to the lack of data, subgroup analysis for infection prevalence in different continents could not be conducted.

The prevalence of leptospirosis in wild cats was reported to be 13.38% (95% CI, 6.25%–21.93%) in serum samples and 15.68% (95% CI, 6.17%–27.96%) in kidney and urine samples, respectively. These samples were used for diagnosis through various methods, with MAT being the most frequently employed method in 41 studies and PCR in seven studies. The MAT and PCR prevalence rates were 12.07% (95% CI, 4.91%–20.94%) and 2.57% (95% CI, 0.00%–22.14%), respectively.

Within the genus subgroup, the highest prevalence of leptospirosis in wild cats was observed in *Puma* spp. at 23.96% (95% CI, 7.05%–44.81%), followed by *Felis* spp. at 14.22% (95% CI, 0.00%–49.67%), *Lynx* spp. at 13.13% (95% CI, 2.22%–28.89%), *Panthera* spp. at 12.79% (95% CI, 2.02%–28.88%) and *Leopardus* spp. at 5.96% (95% CI, 0.00%–21.45%).

#### Meta-regression and cumulative meta-analysis

**Figure-3:**
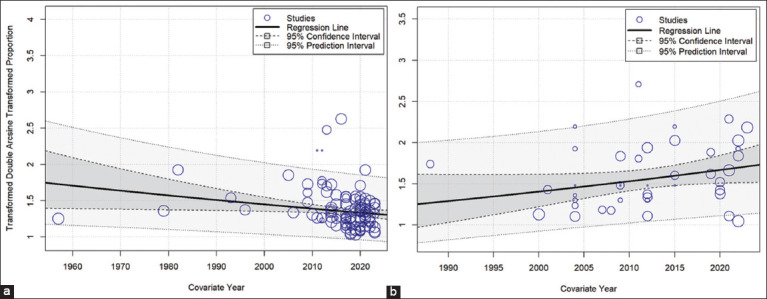
Scatterplot of meta-regression analysis to assess trends in leptospirosis prevalence over the years in (a) domestic cats and (b) wild cats.

Meta-regression analysis revealed a significant association between the moderator variable “Year” and the prevalence of leptospirosis in domestic cats (8.9061–0.0043 * Year [95% CI, 0.0079–0.0007], p = 0.02) (Figure-3). A cumulative meta-analysis showed that the estimated combined prevalence of leptospirosis in domestic cats varied over the years. The prevalence of leptospirosis in domestic cats was 4.86% (95% CI, 2.82%–7.38%) in 1957 and progressively increased to 19.18% (95% CI, 11.03%–28.64%) by 2013. However, since then, the prevalence trend has exhibited a decline, approaching 7.59% (95% CI, 5.61%–9.80%) in 2023.

In contrast, meta-regression analysis did not identify a significant association between the moderator variable “Year” and the prevalence of leptospirosis in wildcats (event rate: 16.0962 + 0.0082 * Year [95% CI, 0.0014–0.0179], p = 0.09) (Figure-3). A cumulative meta-analysis has shown that the estimated combined prevalence of leptospirosis in wild felids fluctuates over time. In 1988, the prevalence of leptospirosis in wildcats was 25% (95% CI: 0.78%–61.53%). Subsequently, it declined to 0% (95% CI, 0.00%–6.10%) by 2000 and gradually increased to 11.19% (95% CI, 7.39%–11.77%) by 2022, with notable variations observed within this period.

### Sensitivity analysis

In the sensitivity analysis conducted for each study, variations in leptospirosis seroprevalence and leptospirosis infection prevalence in domestic and wild cats were observed, although these differences were slight. For domestic cats, the seroprevalence of leptospirosis varied from 9.48% (95% CI, 7.39%–11.77%) to 10.28% (95% CI, 7.97%–12.81%). Similarly, the infection prevalence varied from 3.63% (95% CI, 1.75%–5.99%) to 4.86% (95% CI, 2.25%–8.16%). The seroprevalence of leptospirosis in wildcats also varied, ranging from 12.25% (95% CI, 5.26%–20.80%) to 14.8% (95% CI, 7.48%–23.42%). Similarly, the infection prevalence exhibited slight differences, ranging from 0.41% (95% CI, 0.00%–15.00%) to 5.98% (95% CI, 0.00%–22.97%).

### Risk of publication bias and quality assessment of individual studies

**Figure-4:**
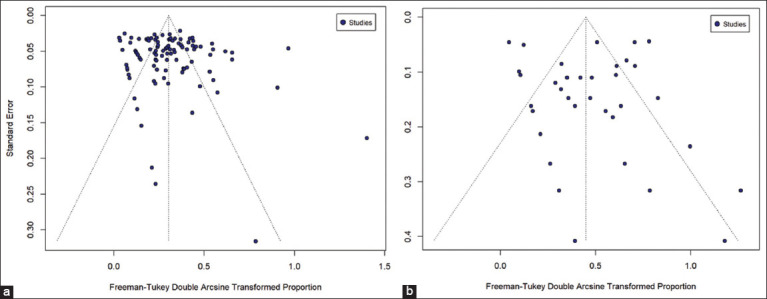
Funnel plots of standard error to assess publication bias across prevalence studies in (a) domestic cats and (b) wild cats.

Funnel plots for both domestic and wildcats, as illustrated in Figure-4, revealed no significant indications of publication bias. Furthermore, the Egger’s test showed no evidence of publication bias between domestic and wildcats (p = 0.08 and p = 0.50, respectively). To assess the risk of bias, 47 studies were found to be of high-quality, whereas 24 studies in the field of domestic cat studies had a moderate quality rating. Wildcat studies comprised 16 high-quality studies and seven studies with moderate-quality ratings (https://osf.io/b3u8j/).

## Discussion

This study emphasized the seroprevalence and infection prevalence of leptospirosis in both domestic and wild cats. Our results reveal that domestic cats exhibited a lower seroprevalence than wildcats (9.95% vs. 13.38%). However, the infection prevalence in domestic cats was higher than in wild cats (4.62% vs. 2.9%). Among wildcats, *Puma* spp. showed the highest prevalence, followed by *Felis* spp., *Lynx* spp., *Panthera* spp., and *Leopardus* spp. in descending order. Several risk factors may contribute to variations in the seroprevalence and infection prevalence of feline leptospirosis, including the proximity of cats to human settlements, residence in flood-prone areas, close interaction with other animals, and the presence of rodents as potential vectors [28, 29].

The prevalence of leptospirosis in domestic cats may vary according to the lifestyle of the cat and geographical location [3, 28, 30]. Domestic cats that have access to the outdoors are susceptible to leptospirosis due to their predilection to prey on rodents, direct engagement with contaminated water sources, and shared habitation with farm animals that serve as potential reservoirs, excreting the leptospiral bacteria in their urine [2]. The transmission of *Leptospira* spp. bacterial infections is linked not only to water sources containing the pathogen but also to the dietary habits of wildcats living in natural environments [102]. Bobcats consume various mammalian prey, including rodents, lagomorphs, white-tailed deer (*Odocoileus virginianus*), and other ungulates [102–104]. The Eurasian lynx (*Lynx lynx*) preys on rodents, ungulates, and European roe deer (*Capreolus capreolus*), with rodents being their alternative food source [105]. It is important to acknowledge that rodents are reservoir hosts for leptospirosis [106], which highlights the potential risk of leptospirosis in wild cats. Furthermore, wildcats living in captivity are suspected to have *Leptospira* spp. infection due to suboptimal hygiene practices [16].

A detailed subgroup analysis at the continental level revealed variations in the 95% CIs for the seroprevalence and infection prevalence of leptospirosis in domestic and wild cats. Notably, our findings underscored that leptospirosis prevalence in Asia is significantly high in both domestic and wildcat populations. This observed pattern of high prevalence reflects human leptospirosis in the Asia-Pacific region, where the disease remains highly endemic with reported incidence rates ranging from 1 to over 10 cases/100,000 individuals [107]. Leptospirosis morbidity is estimated to be high in several Asian countries (especially Southeast and South Asia). For example, India reported an estimated incidence rate of 19.7 cases/100,000 population, while Indonesia reported a rate of 39.2 cases/100,000 population [108].

The prevalence of human leptospirosis is most pronounced in tropical countries, mainly due to conducive social and environmental risk factors that facilitate disease transmission. Major outbreaks of leptospirosis are often associated with factors such as flooding, inadequate sanitation, climate change, and the presence of high-maintenance host populations, including domestic and wild animals. These hosts play a pivotal role in the dissemination of diseases within these regions [107, 109]. As a result, approximately 73% of global leptospirosis cases and fatalities are reported in these tropical regions [108].

The detection of cat leptospirosis can be performed either by direct or indirect methods. Direct methods involve the identification or isolation of *Leptospira* spp. agent from clinical specimens, whereas indirect methods focus on leptospirosis antibody testing [110]. For indirect detection, MAT and serum testing were the most commonly used methods. MAT, in particular, is considered the gold standard for detecting leptospirosis, capable of identifying various serovars of *Leptospira* spp. [111]. Our findings indicate that the cutoff MAT titer values in domestic cats ranged from 1:20 to 1:200, whereas those in wild cats ranged from 1:50 to 1:100. It should be noted that cats usually respond to leptospirosis, both experimentally and in natural infections, with low antibody titers ranging from 1:30 to 1:400 [31]. In view of the fact that leptospirosis vaccines for cats are not currently available, antibodies measured in MAT from cat serum reflect the immune response to *Leptospira* spp. Infection [32].

However, MAT’s utility may be limited during the early stages of the disease due to the lack of specific antibodies in the immune system or low antibody titers, which can lead to false negative results [112]. In addition, the diagnosis of infection can become more complex if the host has been previously exposed by Verma *et al*. [113] and Goris and Hartskeerl [114] to different serogroups. As a result, direct methods provide more favorable results in the early stages of the disease. The most widely utilized direct diagnostic method for detecting cat leptospirosis is PCR using urine samples. This method offers distinct advantages for identifying *Leptospira* spp. during the initial phases of the disease when antibody titers may not be sufficient for indirect methods and during acute leptospirosis. However, PCR using urine samples can be challenging because it is not always practical and often requires catheterization [115]. To improve diagnostic accuracy, it is recommended to combine both direct and indirect evidence methods. For example, PCR-enzyme-linked immunosorbent assay technique can serve as an alternative to MAT, yielding higher accuracy and more reliable results [116].

Meta-regression, which employs a single data point from each study to estimate prevalence trends [117], and cumulative meta-analysis, which utilizes pooled evidence from studies to track the evolution of evidence over time with new research publications [118] offer valuable insights into disease trends. Our meta-regression analysis revealed a significant decrease in the prevalence of leptospirosis in domestic cats over time, whereas wild cats showed no significant trend change over time.

On the basis of a cumulative meta-analysis, the prevalence of leptospirosis in domestic cats was originally reported at 4.86% in 1957. In 2013, this value increased steadily to 19.18% and then decreased to 7.59% in 2023. On the other hand, leptospirosis in wild cats was first reported in 1988 with a prevalence of 25%, which is gradually approaching 11.19% in 2022. This fluctuating trend in feline leptospirosis over time is similar to trends observed in human leptospirosis [119, 120]. In humans, an upward trend was noted during 1997–2012, which was linked to environmental disruptions, such as floods and heavy rainfall, facilitating the dispersion of pathogens across broader areas [119]. This trend reflects an increase in the incidence of leptospirosis in domestic cats in 2013, potentially reflecting increased exposure to pathogens.

Despite this study’s insights, certain limitations warrant recognition. Variations in the number of cat leptospirosis studies between continents raise the possibility of bias in prevalence, particularly in regions with fewer studies, such as Australia and North America. In addition, some categories within subgroups had to be excluded due to a lack of available studies. For this reason, caution should be exercised when interpreting the results of certain subgroup analysis. In line with our primary objective of assessing the overall pooled prevalence of leptospirosis in domestic and wild cats, the variability in serovars was not investigated. We acknowledge the need for further studies to elucidate the specific serovars of *Leptospira* spp. to enhance our understanding of leptospirosis in cats.

## Conclusion

Our study highlights that both domestic and wild cats are susceptible to *Leptospira* spp. bacterial exposure. Although the seroprevalence and infection prevalence of leptospirosis in these feline populations are relatively low, the risk of transmission of *Leptospira* spp. to the surrounding environment should still be considered. Therefore, leptospirosis in domestic and wild cats should be regarded as a public health concern in view of its potential for zoonotic transmission.

## Authors’ Contributions

MA: Conceptualization, methodology, searching databases, screening for inclusion, data extraction, risk of bias assessment, data analysis, and writing-original draft. DMN: Conceptualization, methodology, screening for inclusion, data extraction, risk of bias assessment, and data analysis. SKL: Methodology, searching databases, and writing-review. PpS: Conceptualization and writing-review. BS: Conceptualization. PS: Data analysis and validation. PT: Conceptualization. ST: Conceptualization, methodology, data analysis, validation, and writing-review and editing. All authors have read, reviewed, and approved the final manuscript.
